# Incidence, Distribution, and Lethality of Firearm Injuries in California From 2005 to 2015

**DOI:** 10.1001/jamanetworkopen.2020.14736

**Published:** 2020-08-26

**Authors:** Sarabeth A. Spitzer, Veronica A. Pear, Christopher D. McCort, Garen J. Wintemute

**Affiliations:** 1 Now with the Department of Surgery, Brigham and Women’s Hospital, Boston, Massachusetts; 2 University of California Firearm Violence Research Center, Sacramento; 3 Violence Prevention Research Program, Department of Emergency Medicine, UC Davis School of Medicine, Sacramento

## Abstract

**Importance:**

Little is known about nonfatal firearm injuries in the United States, and national estimates based on emergency department samples may not be accurate.

**Objective:**

To describe the incidence and distribution of nonfatal firearm injuries and estimate case fatality ratios (CFRs) for firearm injuries by external cause of injury code within California overall and by race/ethnicity, including an assessment of trends over time and geographic variation within the state.

**Design, Setting, and Participants:**

This serial cross-sectional study used complete statewide data for firearm-related mortality, emergency department visits, and hospitalizations among California residents from January 1, 2005, through December 31, 2015, to analyze incidence, distribution, and CFRs of firearm injury. Data were analyzed from 2018 to 2019.

**Exposures:**

All individuals in California with a firearm injury based on *International Classification of Diseases, Ninth Revision* or *International Statistical Classification of Diseases and Related Health Problems, Tenth Revision* codes were included.

**Main Outcomes and Measures:**

Counts and rates of nonfatal firearm injuries overall and stratified by external cause, sex, and race/ethnicity; total and clinical CFRs. Clinical CFR was calculated based on individuals treated in emergency departments or hospitals.

**Results:**

Over the study period, there were 81 085 firearm-related emergency department visits and hospitalizations among individuals with a mean (SD) age of 27.5 (11.9) years, 72 567 (89.6%) of whom were men. Nonfatal firearm injuries in California decreased by 38.1% between 2005 and 2015, driven by a 46.4% decrease in assaultive injuries. Self-inflicted injuries and unintentional injuries remained relatively stable. The overall CFR for firearm injuries increased from 27.6% in 2005 to 32.2% in 2015 for a relative increase of 20.7%, while the clinical CFR remained stable between 7.0% and 9.0%.

**Conclusions and Relevance:**

These findings suggest that although the number of firearm injuries has decreased in California, the lethality of these injuries has not. Similar studies from other states could provide more information about these trends nationwide.

## Key Points

**Question:**

What were the trends and distributions of nonfatal firearm injuries and how lethal were firearm injuries in California from 2005 to 2015?

**Findings:**

This serial cross-sectional study including 81 085 firearm-related emergency department visits and hospitalizations found that nonfatal firearm injuries decreased by 38.1% between 2005 and 2015, driven by a 46.4% decrease in assaultive injuries; self-inflicted injuries decreased by 13.4% and unintentional injuries decreased by 12.7%. However, the overall case fatality ratio increased a relative 20.7%, while the clinical case fatality ratio remained stable.

**Meaning:**

These findings suggest that although the number of firearm injuries has decreased in California, the lethality of these injuries has not; studies from other states could help clarify national trends.

## Teaser

This cross-sectional study examines incidence, distribution, and lethality of firearm injuries in California from 2005 to 2015.

## Introduction

Firearm injury is a significant cause of morbidity and mortality in the United States, resulting in more than 350 000 deaths and a far larger number of nonfatal injuries nationwide from 2005 through 2015.^1^ In 2018, firearm-related deaths in the US exceeded those from motor vehicle crashes.^1^ For individuals who survive firearm injuries, the long-term physical and psychological effects can be devastating.^2^ Survivors and their families may face large costs as a result of their injuries, both economically and socially. Total societal costs have been previously estimated to be as high as $229 billion annually and have likely increased.^3^

There are currently only imprecise estimates of the number of annual nonfatal firearm injuries in the US. The accuracy of nonfatal firearm injury estimates by the Centers for Disease Control and Prevention (CDC) have come under scrutiny, sparked by a surprising 37% reported increase in nonfatal injuries from 2015 to 2016, when fatal injuries increased by only 6.6%.^1^ The contrast motivated several research reports regarding the case fatality ratio (CFR) of firearm injury.^4,5,6,7,8,9^ The CDC data, if accurate, would suggest that the lethality of firearm injury is decreasing.^3^ However, this suggestion has been contested by clinicians and researchers alike.^4,5,6,7,8^ The CDC no longer provides estimates of nonfatal firearm assaults for the years 2007 and 2013 to 2018 or of nonfatal self-harm with a firearm for 2001 to 2011 and 2013 to 2018, stating that the estimates are unstable.^1^

California’s statewide enumeration of emergency department (ED) visits and hospitalizations for firearm injuries, coupled with mortality data, offers a unique opportunity to explore the incidence and distribution of nonfatal firearm injury and estimate trends in the CFR over time overall and by external cause of injury (ie, assault, self-inflicted, unintended, and undetermined) codes. A study by Pear and colleagues^10^ previously described the incidence and distribution of firearm mortality in California, but to our knowledge, there are no peer-reviewed studies that explore the incidence and distribution of nonfatal firearm injury in the state. This report complements our previous mortality study^10^; together, given California's size as well as its demographic and geographic diversity, these studies advance our understanding of the incidence, distribution, and lethality of firearm injuries.

## Methods

This study was approved by the University of California, Davis, institutional review board and the California Committee for the Protection of Human Subjects (CPHS). Informed consent was waived per CPHS policy because this study involved no more than minimal risk to participants and data were not identified. This study is reported following the Strengthening the Reporting of Observational Studies in Epidemiology (STROBE) reporting guideline.

This serial cross-sectional study used state-wide data from California’s Office of Statewide Health Planning and Development (OSHPD) for individuals treated in an ED or discharged from a hospital between January 1, 2005, and December 31, 2015. These databases contain all ED and inpatient records from California-licensed hospitals. Additionally, CDC WISQARS data were used for fatal firearm injury data.

We used *International Classification of Diseases, Ninth Revision* (*ICD-9*)^11^ codes E922 (0.0-.3, 0.8, 0.9), E955 (0.0-.4), E965 (0.0-.4), E979.4, E985 (0.0-.4), and E970 to identify all admissions for firearm injuries from 2005 through 2015. Reporting changed from ICD-9 to *International Statistical Classification of Diseases and Related Health Problems, Tenth Revision* (*ICD-10*)^12^ codes in October 2015. Therefore, for the last quarter of 2015, we used initial encounter (A) *ICD-10* codes W32-33, W34 (0.00, 0.09, 0.10, 0.19), X72, X73, X74 (0.8, 0.9), X93, X94, X95 (0.8, 0.9), Y22-3, Y24 (0.8, 0.9), Y35.0, and Y38.4. External cause of injury codes are used to identify admissions related to injury, and these codes correspond to firearm injuries of all causes (eg, assault, self-harm) and all weapon types (eg, handguns, rifles). Owing to small numbers, we grouped codes for terrorism or legal intervention with assaults. We used admission dates to identify firearm injuries; results for 2015 represent a slight undercount because our data did not include injuries for which patients were admitted in 2015 but discharged in 2016. However, we explored the spillover rates for years with complete data and found that less than 1% of patients were admitted in one year and discharged the following year.

To capture only nonfatal injuries, we excluded records with a discharge disposition of death. To avoid double-counting injuries, we excluded records for non–acute care hospitalizations, as these were unlikely to be for new injuries. We also fit a predictive model using Super Learner^13^ to distinguish between acute care cases that were for a new injury and those that were related to a previous injury. Super Learner uses cross-validation to create a single predictive model that minimizes bias by weighting several potential models that are provided by the user.^13^ Model development is described in detail elsewhere.^14^ We excluded records for visits with an Injury Severity Score (ISS) of 0, as this is unlikely to be an acute firearm injury, and those for individuals who were not residents of California. To prevent patient reidentification and in accordance with California state regulations, we removed from our reported results the findings for any study subgroup with fewer than 15 patients.

Other data available from OSHPD included age, sex, payer status, disposition, race/ethnicity, and hospital length of stay. Race/ethnicity was reported as non-Hispanic White, non-Hispanic Black, Hispanic, American Indian, Asian or Pacific Islander, and other. Race and ethnicity were defined by OSHPD and assessed to evaluate epidemiological trends. Standardization of disposition codes across ED and inpatient data can be seen in the eTable in the Supplement.

The US Department of Agriculture Rural-Urban Continuum Codes data were used to determine the urban-rural status of each county. Rural-Urban Continuum Code data distinguish counties based on population and adjacency to metropolitan areas; we collapsed the 9 categories of Rural-Urban Continuum Codes into 2 broader categories of metropolitan (urban) and nonmetropolitan (rural) counties.^15^ We linked this to our OSHPD data by patient county of residency. American Community Survey data were used to determine the median income of zip codes, which we categorized into quartiles. We linked this to patient residential zip codes.

The CDC WISQARS and CDC WONDER databases were used to determine yearly county-level population data, race/ethnicity subpopulation data, and fatal firearm injury data.^1,16^ These values were used as the denominators to create population injury rates and overall CFRs. A verified Stata module (StataCorp), ICD-PIC, was used to translate *ICD-9* codes into standard Injury Severity Scores (ISSs).^17^ ICDPICR, a tool translating ICD-PIC into an R package (R Project for Statistical Computing), was used to translate *ICD-10* codes into standard ISS.^18^

The primary outcome measures were counts and rates of nonfatal firearm injuries and the overall and clinical CFRs of firearm injuries in California. Counts and rates were described over time and grouped by external cause.

### Statistical Analysis

The overall CFR was calculated by dividing all firearm deaths in California as measured by WISQARS by the total number of firearm injuries (WISQARS fatal + OSHPD nonfatal) per year. The clinical CFR was calculated by dividing the number of firearm fatalities in the OSHPD data (both ED and hospital inpatients) by the total number of firearm injuries (fatal + nonfatal) in the OSHPD data.

County-level rates of nonfatal injury in California were mapped to show the geographic distribution of firearm morbidity. To account for the small numbers and concomitant unstable rates in some counties, we used a random-intercept Poisson mixed-effects model to smooth the rates, with random effects for year and county, as well as an offset for the log-population. These smoothed rates were then used to map the geographic distribution of nonfatal firearm injuries in California by county. Negative binomial regressions that included the counts of firearm injuries per county per year and a binary urban-rural variable were used to determine the significance of urbanicity on firearm injuries.

All rates of change and percentage changes over the study period were calculated using generalized linear (Poisson for injury rates, binomial for CFR) mixed-effects models with a linear fixed effect for time incorporated into each to more robustly estimate significant changes over our study years, reported as percentage change in model mean, instead of merely reporting the end points. All rates are reported per 100 000 residents of the relevant population.

We used *t* tests for continuous data and χ^2^ tests to compare categorical variables. We considered 2-sided *P* < .05 to be significant. R version 3.4.4 with R Studio version 1.1.453 (RStudio) and Stata SE version 14.1 were used for analyses. Data were analyzed from 2018 to 2019.

## Results

A total of 81 085 nonfatal firearm injuries were identified from 2005 through 2015, including 56 367 assaultive injuries (69.7%), 19 316 unintentional injuries (23.6%), 1372 self-inflicted injuries (1.7% ), and 4030 injuries of undetermined intent (5.0%) (Table). The mean (SD) age of individuals with firearm injuries was 27.5 (11.9) years, and 72 567 (89.6%) were men. A total of 45 570 injuries (56.2%) were treated within the ED and did not include hospital admission, while 35 515 injuries (43.8%) included admission to an inpatient facility. Those with assaultive injuries tended to be younger (mean [SD] age, 26.8 [10.7] years) and Black (18 355 patients [33.3%]) or Hispanic (25 423 patients [46.1%]), while those with self-inflicted injuries were more likely to be older (mean (SD) age, 42.3 [18.6] years) and White (817 patients [62.2%]). There were differences in income and payment source by cause of injury as well: individuals with assaultive injuries, compared with those with self-inflicted injuries, were more likely to be within the lowest income quartile (16 081 patients [29.5%] vs 225 patients [16.4%]) and have self-pay (18 553 patients [32.9%] vs 300 patients [21.9%]) or government (20 852 patients [37.0%] vs 322 patients [23.5%]) payer status. Individuals with injuries from self-inflicted gunshot wounds had worse markers for increased severity compared with other injury causes, including higher median (interquartile range) ISS (self-inflicted: 9 [1-16]; assaultive: 4.0 [2-9]; unintentional: 4.0 [1-7]; undetermined: 3.0 [1-7]; *P* < .001), longer median (interquartile range) length of stay (self-inflicted: 8.0 [3-17] days; assaultive: 4.0 [2-9] days; unintentional: 4.0 [1-7] days; undetermined: 3.0 [1-7] days; *P* < .001), and a smaller proportion of routine discharges to home (self-inflicted: 502 patients [36.6%]; assaultive: 46 034 patients [81.7%]; unintentional: 15 830 patients [82.0%]; undetermined: 3212 patients [79.7%]; *P* < .001).

**Table.** Demographic Characteristics Among Survivors of Nonfatal Firearm Injuries by e-Coded External Cause From 2005 to 2015

Characteristic	Assault (n = 56 367)	Self-inflicted (n = 1372)	Unintentional (n = 19 316)	Undetermined (n = 4030)	Total (N = 81 085)	*P* value
Age, y^a^						
Mean (SD)	26.8 (10.7)	42.3 (18.6)	28.9 (13.8)	26.5 (11.1)	27.5 (11.9)	<.001
Median (IQR)	24.0 (19-32)	41.0 (26-55)	24.0 (19-35)	23.0 (19-32)	24.0 (19-33)	
Sex						
Women	5513 (9.8)	248 (18.1)	2217 (11.5)	416 (10.4)	8394 (10.4)	<.001
Men	50 753 (90.2)	1124 (81.9)	17 087 (88.5)	3603 (89.6)	72 567 (89.6)	
Payer status						
Medicare	1383 (2.5)	213 (15.5)	825 (4.3)	115 (2.9)	2536 (3.1)	<.001
Government—low income	20 852 (37.0)	322 (23.5)	5263 (27.2)	1263 (31.3)	27 700 (34.2)	
Private or work-based	14 752 (26.2)	519 (37.8)	6406 (33.2)	1015 (25.2)	22 692 (28)	
Self-pay	18 553 (32.9)	300 (21.9)	6563 (34)	1563 (38.8)	26 979 (33.3)	
Other, not reported, or invalid	826 (1.5)	18 (1.3)	259 (1.3)	74 (1.8)	1177 (1.5)	
Disposition						
Routine	46 034 (81.7)	502 (36.6)	15 830 (82.0)	3212 (79.7)	65 578 (80.9)	<.001
Inpatient care transfer	5089 (9)	573 (41.8)	2043 (10.6)	502 (12.5)	8207 (10.1)	
Skilled nursing or resident care facility	397 (0.7)	56 (4.1)	106 (0.5)	23 (0.6)	582 (0.7)	
Intermediate care	139 (0.2)	5 (0.4)	35 (0.2)	6 (0.1)	185 (0.2)	
Children’s hospital or cancer center	65 (0.1)	7 (0.5)	42 (0.2)	4 (0.1)	118 (0.1)	
Against medical advice	1004 (1.8)	10 (0.7)	363 (1.9)	82 (2)	1459 (1.8)	
Law enforcement or prison	1651 (2.9)	36 (2.6)	291 (1.5)	72 (1.8)	2050 (2.5)	
Other	1988 (3.5)	183 (13.3)	606 (3.1)	129 (3.2)	2906 (3.6)	
Race/ethnicity						
White	7456 (13.5)	817 (62.2)	4991 (26.8)	645 (16.6)	13 909 (17.6)	<.001
Black	18 355 (33.3)	85 (6.5)	4623 (24.8)	1258 (32.5)	24 321 (30.8)	
Hispanic	25 423 (46.1)	316 (24.1)	7657 (41.2)	1662 (42.9)	35 058 (44.4)	
Asian or Pacific Islander	1665 (3)	36 (2.7)	595 (3.2)	145 (3.7)	2441 (3.1)	
Native American, Alaska Native	145 (0.3)	4 (0.3)	90 (0.5)	18 (0.5)	257 (0.3)	
Other	2099 (3.8)	55 (4.2)	651 (3.5)	147 (3.8)	2952 (3.7)	
Unspecified weapon type	38 260 (67.9)	548 (39.9)	13 765 (71.3)	3319 (82.4)	55 892 (68.9)	<.001
Income quartile						
0-25th	16 081 (28.5)	225 (16.4)	4361 (22.6)	887 (22)	21 554 (26.6)	<.001
25-50th	14 947 (26.5)	278 (20.3)	4910 (25.4)	1183 (29.4)	21 318 (26.3)	
50-75th	14 573 (25.9)	382 (27.8)	5331 (27.6)	1106 (27.4)	21 392 (26.4)	
75-100th	10 759 (19.1)	487 (35.5)	4700 (24.3)	854 (21.2)	16 800 (20.7)	
Residence						
Metropolitan	55 992 (99.3)	1279 (93.2)	18 712 (96.9)	3970 (98.5)	79 953 (98.6)	<.001
Nonmetropolitan	375 (0.7)	93 (6.8)	604 (3.1)	60 (1.5)	1132 (1.4)	
Length of stay, median (IQR), d^b^	4.0 (2-9)	8.0 (3-17)	4.0 (1-7)	3.0 (1-7)	4.0 (2-8)	<.001
Injury Severity Score, median (IQR)	4 (1-9)	9 (1-16)	1 (1-4)	5 (1-5)	6 (1-9)	<.001

Abbreviation: IQR, interquartile range.

^a^ Does not include individuals aged 100 years or older.

^b^ Measured only for those individuals who were admitted, not those released from the emergency department.

**Figure 1.**
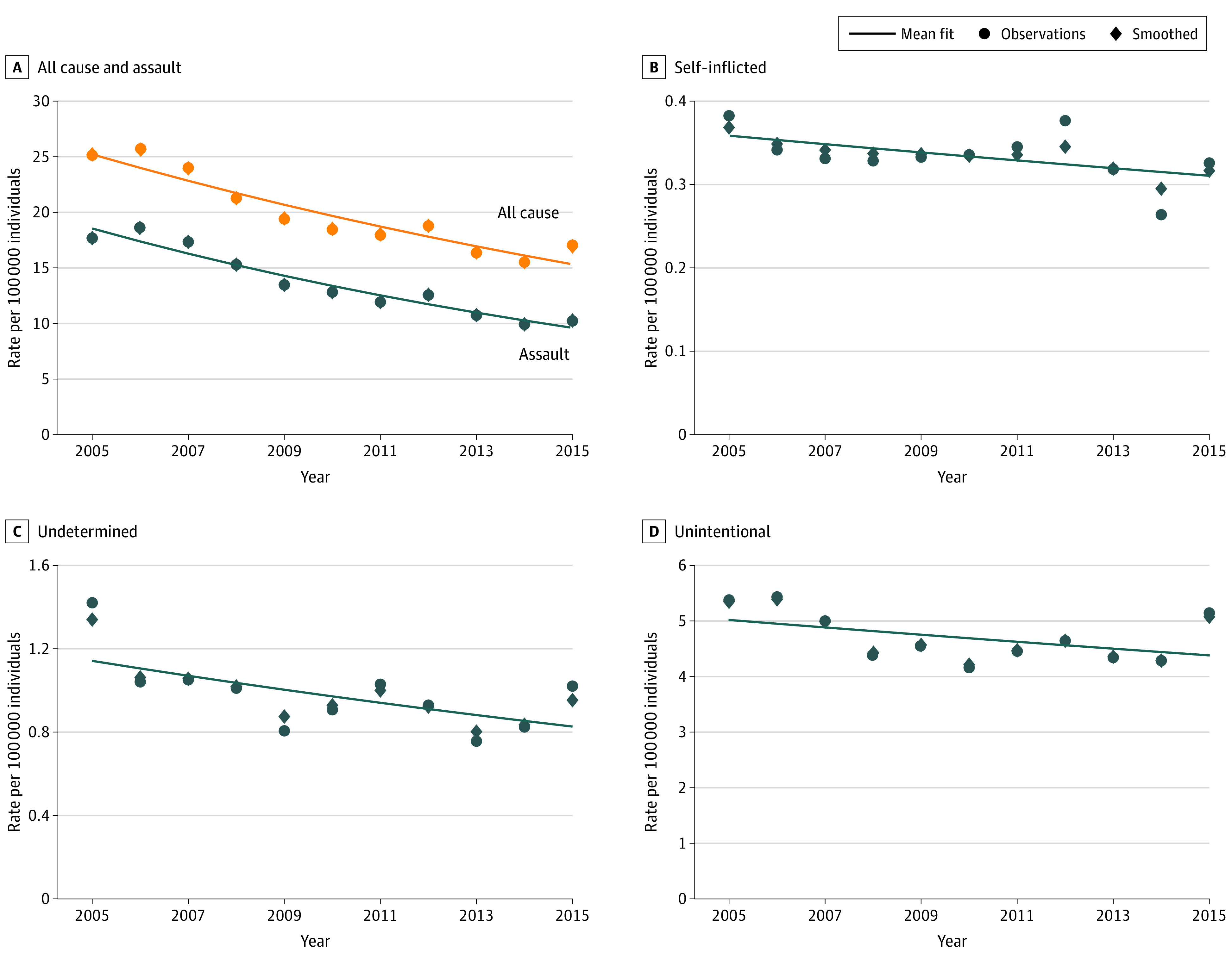
Annual Rate of Nonfatal Firearm Injury per 100 000 People From 2005 to 2015

The overall rate of nonfatal firearm injuries decreased by 38.1% from 2005 through 2015, driven primarily by a 46.4% decrease in assaults (Figure 1). Self-inflicted and unintentional injuries remained stable.

Among men, the overall rate of nonfatal firearm injuries decreased from 45.2 per 100 000 people to 30.2 per 100 000 people from 2005 through 2015, driven primarily by a decrease in assaults of nearly 50%. The rate of self-inflicted and unintentional injuries among men remained stable over the period. Similar trends can be seen for women, although on a much smaller scale; firearm injury rates among women were significantly lower than among men (eFigure 1 in the Supplement). This makes it difficult to assess subcategories of firearm injury among women, such as by race/ethnicity.

**Figure 2.**
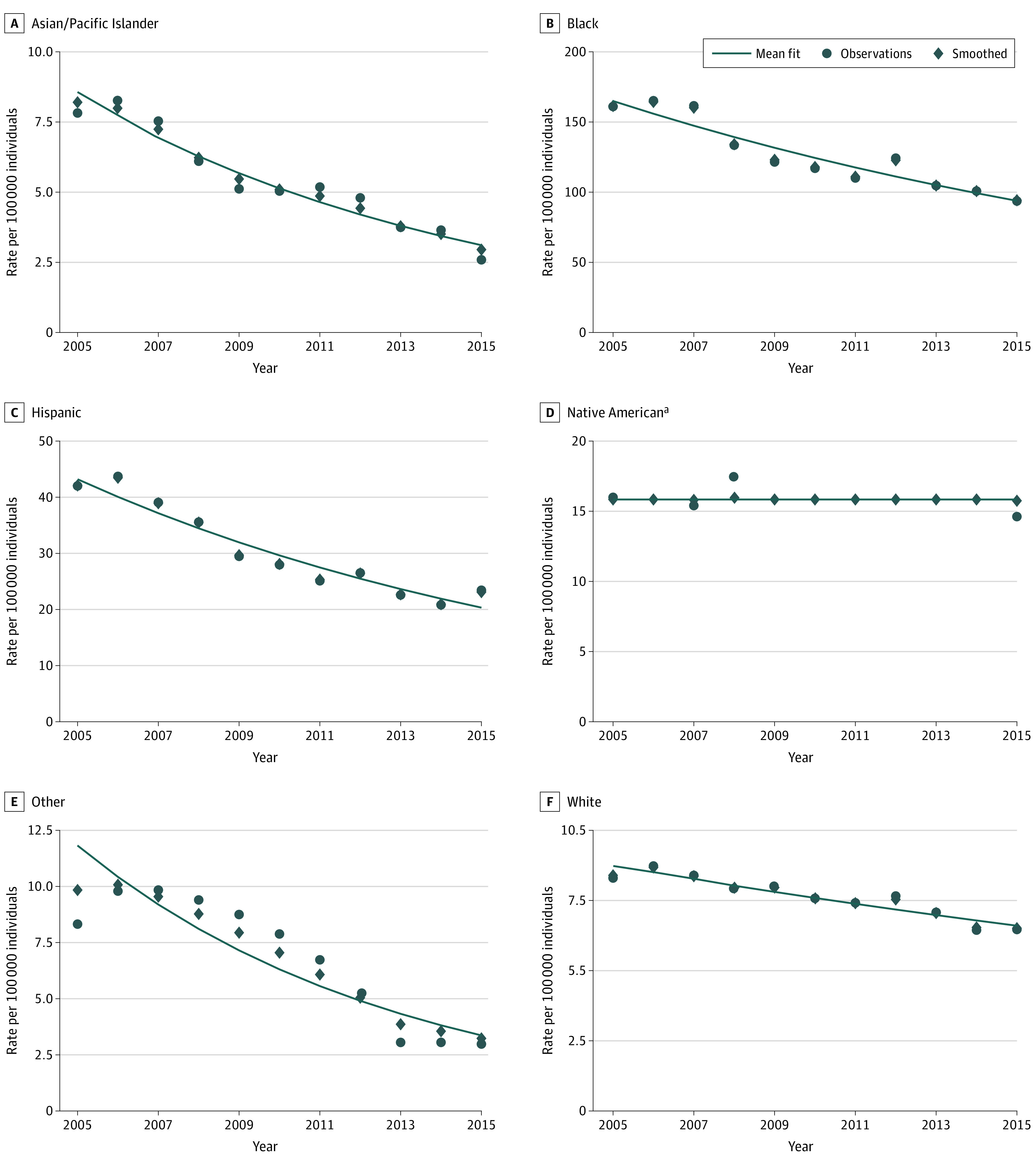
Annual Rate of Assaultive Nonfatal Firearm Injuries per 100 000 People Among Men From 2005 to 2015 ^a^Insufficient unsuppressed observations to estimate slope.

Overall, Black men had an annual firearm assault injury rate of 126.5 per 100 000 people, 4-fold that of Hispanic men, the racial/ethnic group with the next highest rate (30.6 per 100 000 people). Assaultive firearm injuries among Black men decreased from 161.1 per 100 000 people to 94.2 per 100 000 people over the study period. The rate among Hispanic men decreased from 42.0 per 100 000 people to 23.4 per 100 000 people, for a relative decrease of 52.9% (Figure 2).

Black men had the highest rate of unintentional nonfatal firearm injuries, with a slight increase over the study period from 30.2 per 100 000 people to 34.6 per 100 000 people. In contrast, Hispanic men had an 18.8% modeled relative decrease in unintentional firearm injuries. The rate among White men was stable. (eFigure 2 in the Supplement).

Native American data are reported where appropriate per our methods and otherwise suppressed. Trends for women and for both sexes were similar as those presented for men but on a much smaller scale (eFigure 3 in the Supplement).

### CFRs

The model-smoothed overall CFR increased from 27.6% in 2005 to 32.2% in 2015, for a relative increase of 20.7% (eFigure 4 in the Supplement). The overall CFR for assaultive firearm injuries increased from 23.3% to 26.6%, while that for self-inflicted injuries was stable and remained greater than 90% each year in the study period. The overall CFR for unintentional injuries decreased from 5.3% to 1.1% (modeled relative decrease, 77.0%).

**Figure 3.**
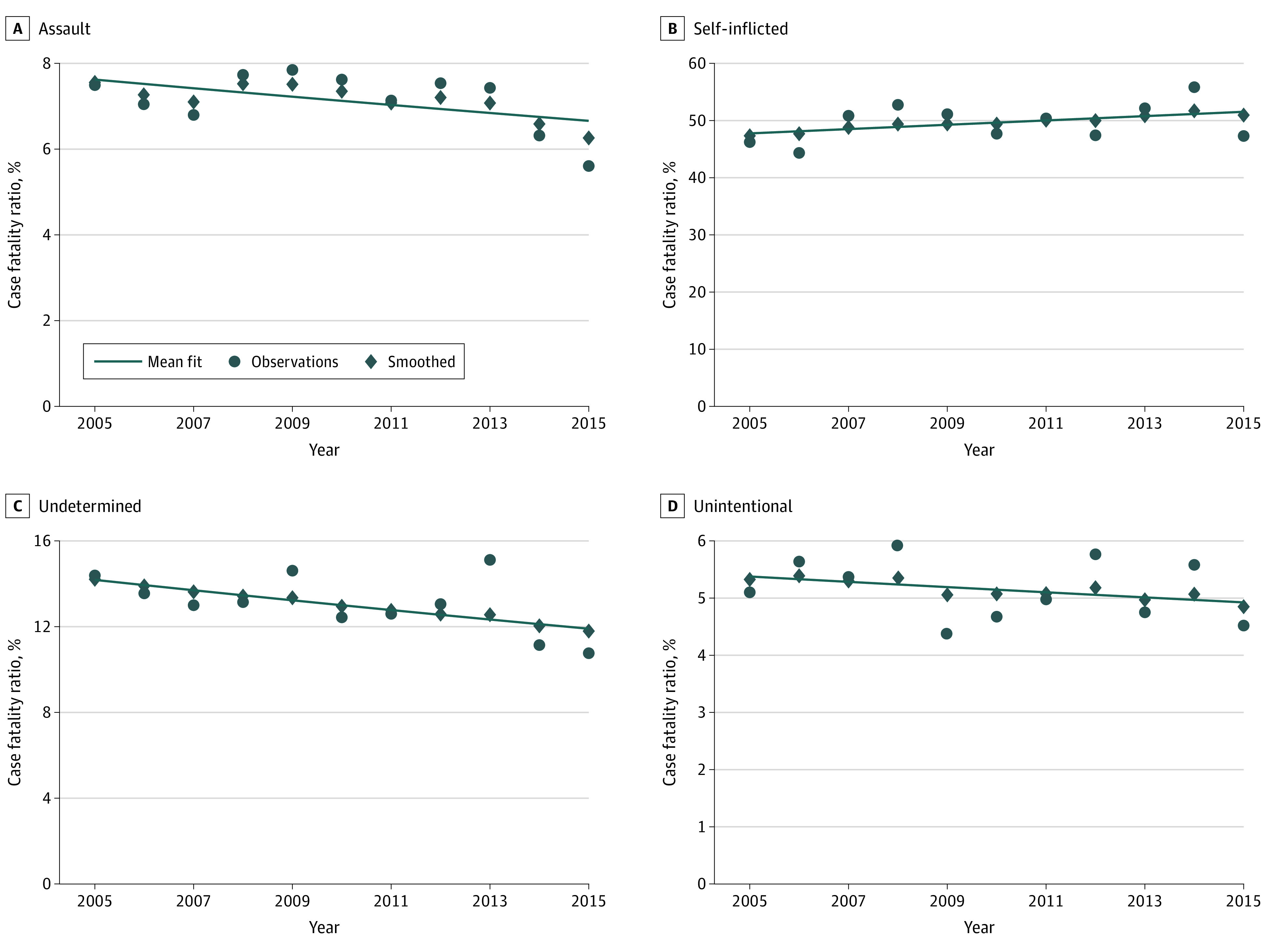
Clinical Firearm Case Fatality Ratio by External Cause From 2005 to 2015

While the clinical CFR did not change significantly over the study period for all injuries combined, there was a significant decrease in the clinical CFR for assault injuries by 1.5%. (Figure 3).

### Geographic Variation

**Figure 4.**
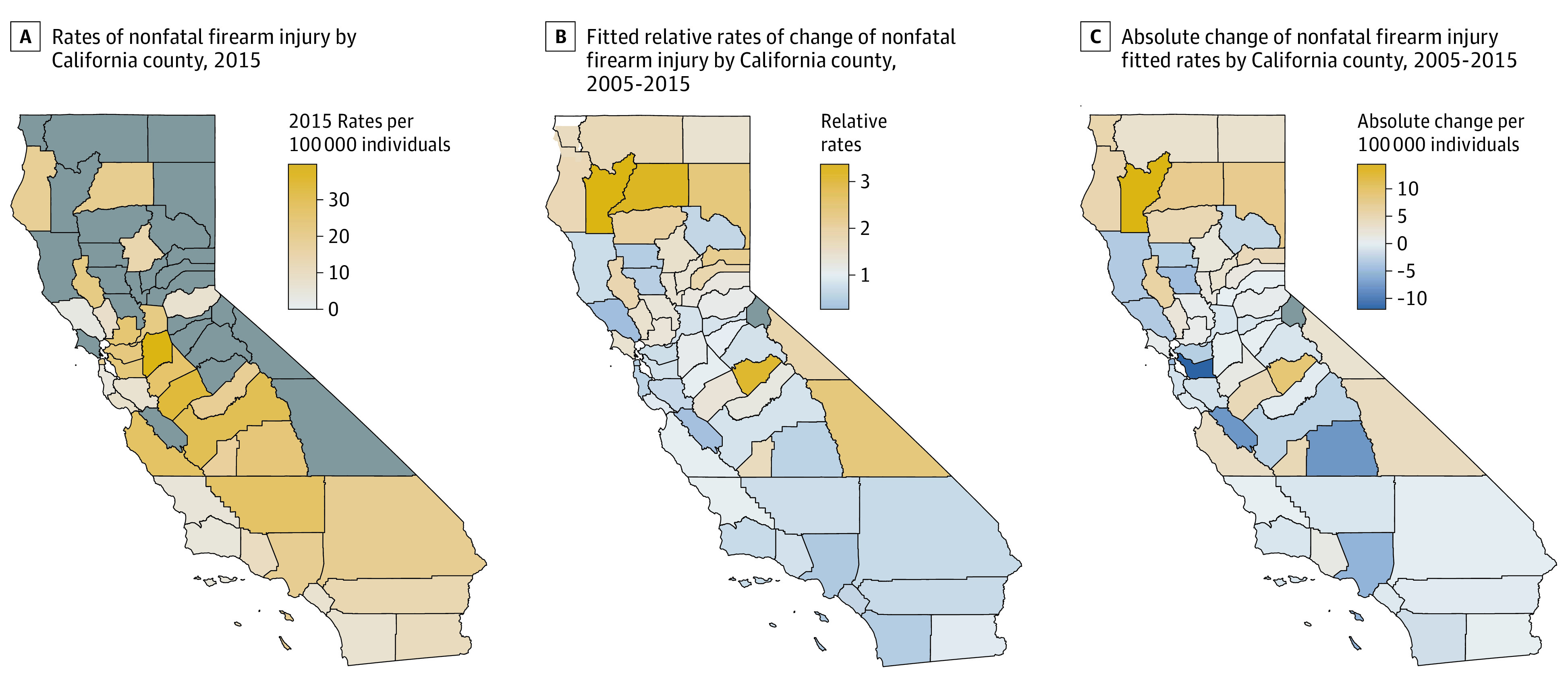
Rates of Nonfatal Firearm Injury by California County in 2015 Counties with fewer than 15 firearm injuries were suppressed.

The smoothed rates of nonfatal injury by county varied substantially in 2015, from a high of 39.7 injuries per 100 000 people in San Joaquin County to a low of 3.6 injuries per 100 000 people in Sonoma County (Figure 4A). Alpine County was suppressed owing to small population and insignificant trends. We also found a significantly increased rate of nonfatal firearm injury in urban relative to rural counties (incidence rate ratio, 1.40; 95% CI, 1.00-1.95).

Sonoma and Los Angeles counties had the largest relative decrease in firearm injuries, at 73.8% in Sonoma County and 58.2% in Los Angeles County (Figure 4B). Of California’s 58 counties, 28 (48.3%) experienced a decrease in the rate of nonfatal firearm injury during the study period. Counties with rate increases tended to be in Northern California. Absolute changes in fitted rates are reported in Figure 4C.

## Discussion

This serial cross-sectional study found that nonfatal firearm injuries in California decreased by nearly 40% from 2005 to 2015, driven primarily by a decrease in assaults across all racial/ethnic groups and sexes, although the difference was most pronounced among Black men.

 The demographic distribution of patients was consistent with known epidemiological patterns in firearm injuries, with rates much higher for men than women, assaultive injuries concentrated among young Black and Hispanic individuals from urban, lower-income areas, and self-inflicted injuries concentrated among White individuals in higher-income areas.^10,19^ As expected, ISSs and hospital length of stay were higher for self-inflicted injuries than for other injury causes. We found that urban counties had higher rates of firearm injury than their rural counterparts, with the highest rates seen in the San Joaquin Valley in central California.

From 2005 to 2015, California’s overall CFR for firearm injuries increased by more than 20% in relative terms. This increase was partially driven by an increase in the proportion of self-inflicted injuries, which are more lethal than assaults; even so, the CFR for assaults also increased by nearly 15% in relative terms, especially in the most recent year of data. This is consistent with other literature examining CFRs of firearm injury^20,21^ and may be explained by an increase in nonsurvivable assaultive injuries. This is contrary to unintentional injuries, for which the overall CFR decreased significantly during the study period.

Despite the increase in overall CFR, clinical CFR remained relatively stable. This discrepancy suggests an increase in the proportion of individuals with fatal injuries who did not reach the ED or hospital to be treated. Researchers have offered at least 2 possible explanations for the stable clinical CFR. One is that injury severity among patients who receive acute medical care has increased, such that improved care has not reduced mortality. However, our data suggest that injury severity has not increased over the study period. The second and more likely explanation in the context of these data is that, in California and during our study period, treatment of patients with life-threatening firearm injuries who reach the hospital has remained stable.

However, findings from a 2020 study by Tessler et al^9^ of injuries from firearms and motor vehicle crashes suggest that the first hypothesized explanation is correct: given that the CFR for motor vehicle crash injuries decreased while that for firearm injuries did not, and assuming that firearm and motor vehicle crash injuries receive the same level of care, there is evidence for there being an increase in firearm injury severity. Tessler et al reported that, except for firearm suicide, ISSs for firearm and motor vehicle crash injuries remained stable over their study period. They suggested that ISS might not be sensitive enough to detect changes in true severity. If this is true, it is also possible that the severity of motor vehicle crash injuries is subtly decreasing. Alternatively, trauma care for motor vehicle crash injuries and firearm injuries might not be improving at equivalent rates.

One strength of this study is that it relies on a complete enumeration of nonfatal injuries. Such data are not often available. Our CFR findings contradict the findings of a study by Kalesan et al^4^ that relied on the CDC’s national estimates for nonfatal injuries and suggested that there was a “hidden epidemic” of nonfatal firearm assaults. Work by our group^5,8^ and others^6,7^ suggests these findings may be invalid.

The findings of this study suggest more research is needed to determine why the overall and cause-specific CFRs did not decrease. It is possible that the wounds are simply not survivable. To explore these questions, further studies to determine trends over time in preventable deaths among individuals who reach level 1 trauma centers are needed. However, over the study period, only 25.2% of deaths were found in the OSHPD data; the rest never reached the ED.

It is well known that most firearm-related deaths occur in the field.^22^ This might make a case for faster or improved transport and further study of the practices of emergency responders, such as the practice of “scoop and run” that is routine in Philadelphia.^23^ Most directly, this makes the case for improved primary prevention efforts, such as discussing firearms with patients who are at risk for harm to self and others^24^ and more effective violence prevention policies, and secondary prevention efforts, such as hospital-based violence prevention programs.^25^

### Limitations

This study has some limitations, the most important of which is that its data are for a single state, limiting generalizability. However, state data are needed because policy efforts to prevent firearm-related violence are primarily enacted at the state level. The US Congress has not enacted major changes to firearm policy in decades.

Additionally, reliance on *ICD-9* and *ICD-10* codes to capture firearm injuries is predicated on accurate and complete coding; miscoded firearm injuries are missed in this data set. In addition, the switch from *ICD-9* to *ICD-10* codes could introduce a change in capture rate of firearm injury in the last quarter of 2015. Third, self-inflicted injuries represent a very small percentage of nonfatal injuries given their high CFR, making it difficult to draw conclusions regarding trends from these data. Fourth, 5% of nonfatal injuries had an undetermined intent, and weapon type was missing in 69% of all injuries, making the weapon type unsuitable for analysis.

## Conclusions

The results of this cross-sectional study could help clarify trends in the incidence and distribution of nonfatal firearm injury and the lethality of firearm injury in California. The results may be valuable to policy makers, public health professionals, clinicians, and researchers as they better tailor clinical practice and public policy to prevent firearm injuries and deaths. We hope this analysis will act as a model for other states, and we wish to emphasize the importance of access to statewide data for researchers in completing similar studies. The conjunction of multiple state-based analyses would allow us to come to a better understanding of nonfatal firearm injuries, which result in substantial burden to individuals, communities, and society at large.

## Notes

Supplement. **eTable.** Defining Disposition **eFigure 1.** Annual Rate of Nonfatal Firearm Injury Per 100 000 People in 2005-2015 Stratified by Sex **eFigure 2.** Annual Rate of Unintentional Nonfatal Firearm Injuries Per 100 000 People Among Men **eFigure 3.** Annual Rate of Assaultive and Unintentional Nonfatal Firearm Injuries Per 100 000 People Among Women **eFigure 4.** Overall Firearm Case Fatality Ratio by External Cause From 2005 to 2015
