# Supplementary material for: Incidence, Distribution, and Lethality of Firearm Injuries in California From 2005 to 2015
Source: JAMA Netw Open. 2020 Aug 26;3(8):e2014736. doi: 10.1001/jamanetworkopen.2020.14736 (PMC7450357; doi:10.1001/jamanetworkopen.2020.14736)
Supplement: Supplement. — eTable. Defining Disposition eFigure 1. Annual Rate of Nonfatal Firearm Injury Per 100 000 People in 2005-2015 Stratified by Sex eFigure 2. Annual Rate of Unintentional Nonfatal Firearm Injuries Per 100 000 People Among Men eFigure 3. Annual Rate of Assaultive and Unintentional Nonfatal Firearm Injuries Per 100 000 People Among Women eFigure 4. Overall Firearm Case Fatality Ratio by External Cause From 2005 to 2015 [file jamanetwopen-3-e2014736-s001.pdf]

Supplementary Online Content

Spitzer SA, Pear VA, McCort CD, Wintemute GJ. Incidence, distribution, and lethality of firearm injuries in California from 2005 to 2015. *JAMA Netw Open*. 2020;3(8):e2014736. doi:10.1001/jamanetworkopen.2020.14736

eTable. Defining Disposition
eFigure 1. Annual Rate of Nonfatal Firearm Injury Per 100 000 People in 2005-2015 Stratified by Sex
eFigure 2. Annual Rate of Unintentional Nonfatal Firearm Injuries Per 100 000 People Among Men
eFigure 3. Annual Rate of Assaultive and Unintentional Nonfatal Firearm Injuries Per 100 000 People Among Women
eFigure 4. Overall Firearm Case Fatality Ratio by External Cause From 2005 to 2015

This supplementary material has been provided by the authors to give readers additional information about their work.

© 2020 Spitzer SA et al. *JAMA Network Open.*

eTable. Defining Disposition

**Disposition**	**Inpatient Disposition Categories 2006-2014**	**Inpatient Disposition Categories 2015**	**ED Dispoition Categories 2006-2009**	**ED Dispoition Categories 2010-2015**
Routine	1 "Routine"	1 "Routine"	1 "Routine"	1 "Routine"
Inpatient care transfer	2 "Acute care within hospital"	2 "Inpatient care transfer"	2 "Inpatient care transfer"	2 "Inpatient care transfer"
	3 "Other care"	82 "Inpatient Hospital with planned readmission"	61 "Medicare swing bed, hospital"	61 "Medicare swing bed, hospital"
	5 "Acute care transfer"	61 "Medicare swing bed, hospital"	66 "Critical access hospital"	66 "Critical access hospital"
	6 "Other care transfer"	66 "Critical access hospital"	62 "Inpatient rehab"	69 "Disaster alternative care site"
		62 "Inpatient rehab"		82 "Short term hosp with planned readm"
				89 "Medicare swing with planned read"
				94 "CAH with planned read"
				62 "Inpatient rehab"
Skilled nursing, res. care facility	7 "Skilled nursing at another facility"	3 "SNF with Medicare"	3 "SNF with Medicare"	3 "SNF with Medicare"
	8 "Residential care facility"	43 "Federal health care facility"	43 "Federal health care facility"	43 "Federal health care facility"
		64 "SNF, Medicaid"	63 "Long term care hospital, medicare"	63 "Long term care hospital, medicare"
		92 "Medicaid nursing facility"	64 "SNF, Medicaid"	64 "SNF, Medicaid"
		63 "Long term care hospital, medicare"		83 "SNF with planned read"
				84 "SNF with planned read"
				88 "Federal health facility with planned read"
				91 "Long term care hospital with planned read"
				92 "Nursing Medicare with planned read"
Intermediate care				93 "Nursing Medical with planned read"

© 2020 Spitzer SA et al. *JAMA Network Open.*

Disposition	Inpatient Disposition Categories 2006-2014	Inpatient Disposition Categories 2015	ED Dispoition Categories 2006-2009	ED Dispoition Categories 2010-2015
Childrens/ cancer	4 "Skilled nursing, intermediate care"	4 "Intermediate Care Facility"	4 "Intermediate Care Facility"	4 "Intermediate Care Facility"
		5 "Children's hospital, cancer center"		5 "Cancer center, children's"
AMA				85 "Cancer/ childrens with planned read"
Law enforcement/prison	10 "AMA"	7 "AMA"	7 "AMA"	7 "AMA"
	9 "Law enforcement"	21 "Law enforcement"	21 "Law enforcement"	21 "Law enforcement"
Other		87 "Law enforcement with planned readmission"		87 "Court/ law enforcement with planned read"
	13 "Other"	0 "Other"	0 "Other"	0 "Other"
		70 "Transfer to other"	5 "Other"	70 "Other"
			70 "Other"	95 "Other with planned read"
		50 "Hospice at home"	50 "Hospice at home"	50 "Hospice at home"
		51 "Hospice at facility"	51 "Hospice at facility"	51 "Hospice at facility"
	12 "Home health"	6 "Home with organized care"	6 "Home with organized care"	6 "hHme with organized care"
		81 "Home with Planned readmission"		81 "Home with planned readm"
				86 "Home health with planned read"
		65 "Psychiatric hospital"	65 "Psychiatric hospital"	65 "Psychiatric hospital"
		93 "Psychiatric admission with planned read"		
Invalid/ Blank, missing [excluded]	0 "Invalid/blank"	99 "Invalid/ blank"	99 "Invalid/ blank"	99 "Invalid/ blank"
Died [excluded]	11 "Died"	20 "Died"	20 "Died"	20 "Died"

The inclusion criteria for “Disposition” as used in Table 1 as defined by OSHPD inpatient and ED disposition information.

© 2020 Spitzer SA et al. *JAMA Network Open.*

eFigure 1. Annual Rate of Nonfatal Firearm Injury Per 100 000 People in 2005-2015 Stratified by Sex

eFigure 1a

Annual Rate of Nonfatal Firearm Injury Per 100 000 Among Men, 2005-2015

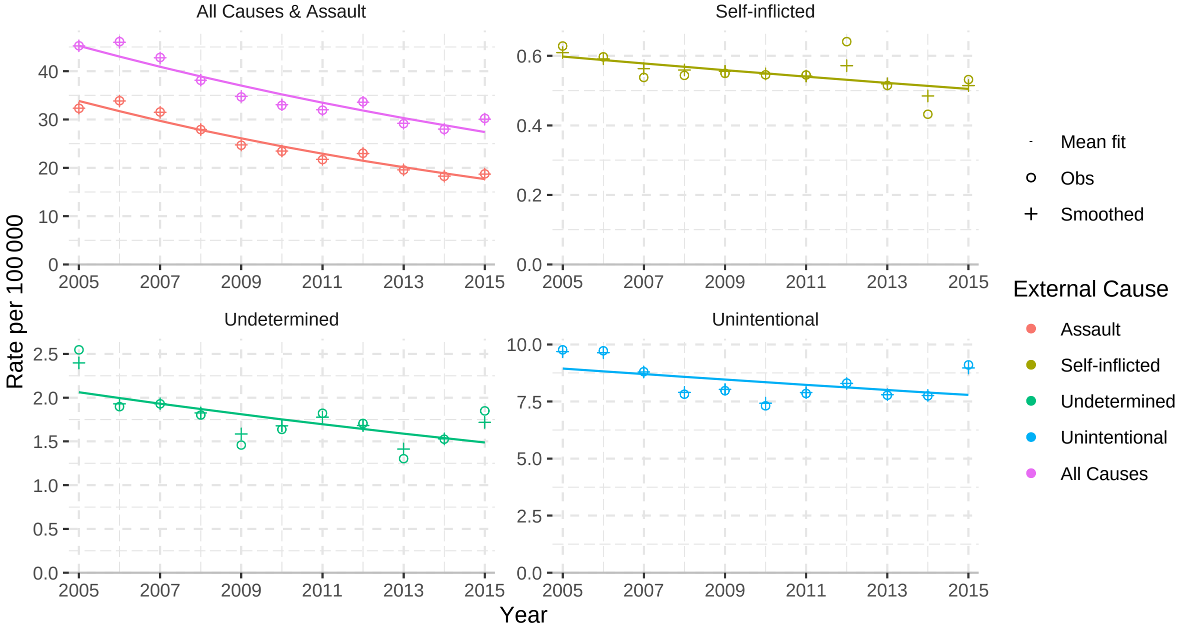

The annual rate of nonfatal firearm injury among men per 100,000 between 2005 and 2015 by the external cause code of injury.

eFigure 1b

Annual Rate of Nonfatal Firearm Injury Per 100 000 Among Women, 2005-2015

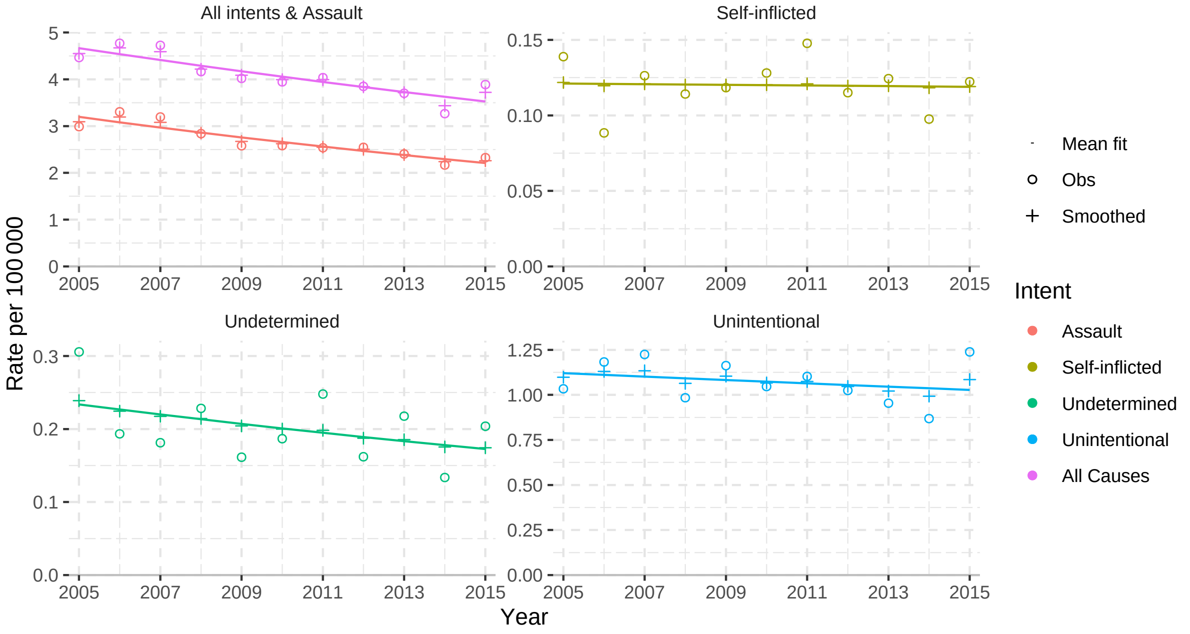

The annual rate of nonfatal firearm injury among women per 100,000 between 2005 and 2015 by the external cause code of injury.

© 2020 Spitzer SA et al. *JAMA Network Open.*

eFigure 2. Annual Rate of Unintentional Nonfatal Firearm Injuries Per 100 000 People Among Men

eFigure 2

Annual Rate of Unintentional Nonfatal Firearm Injuries Per 100 000 Among Men

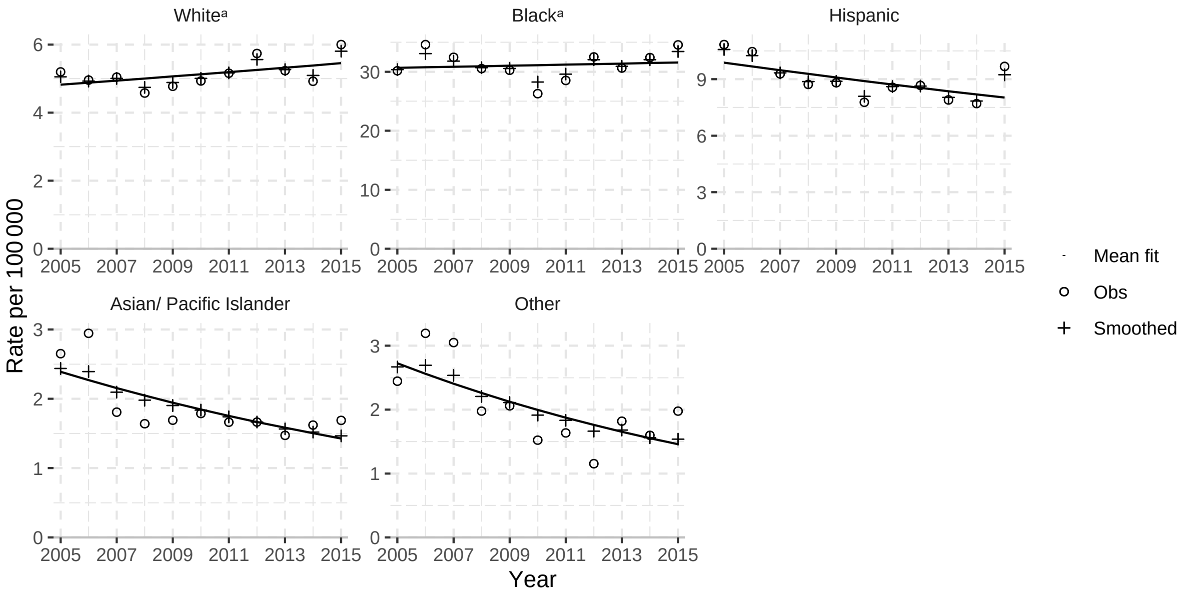

The annual rate of male unintentional nonfatal firearm injury per 100,000 between 2005 and 2015 by race.

[a]slope NS

Native American suppressed

© 2020 Spitzer SA et al. *JAMA Network Open.*

eFigure 3. Annual Rate of Assaultive and Unintentional Nonfatal Firearm Injuries Per 100 000 People Among Women

eFigure 3a

Annual Rate of Assaultive Nonfatal Firearm Injuries Per 100 000 Among Women

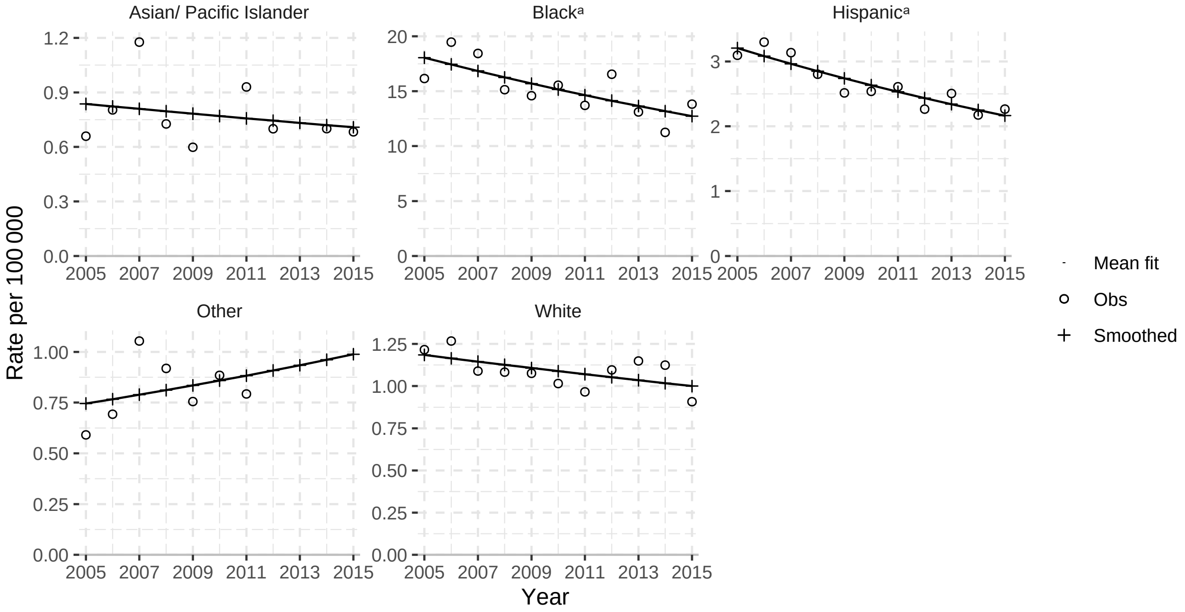

The annual rate of female assaultive nonfatal firearm injury per 100,000 between 2005 and 2015 by race.
[a]p-value for slope < 0.05

© 2020 Spitzer SA et al. *JAMA Network Open.*

eFigure 3b

Annual Rate of Unintentional Nonfatal Firearm Injuries Per 100 000 Among Women

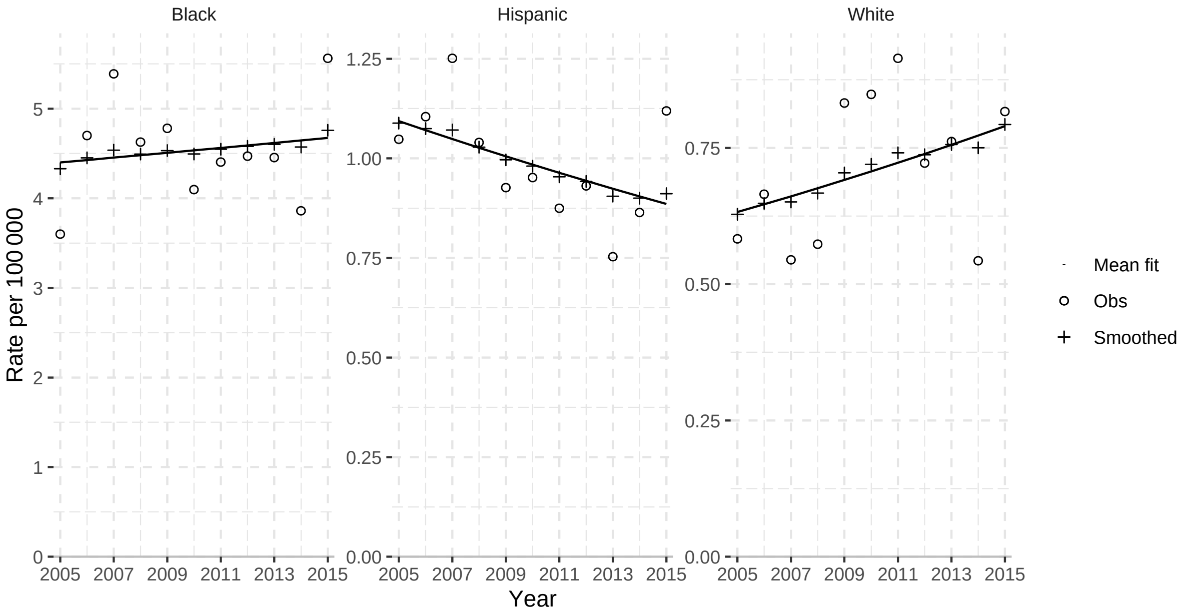

The annual rate of female unintentional nonfatal firearm injury per 100,000 between 2005 and 2015 by race.
Slopes N.S.

© 2020 Spitzer SA et al. *JAMA Network Open.*

eFigure 4. Overall Firearm Case Fatality Ratio by External Cause From 2005 to 2015

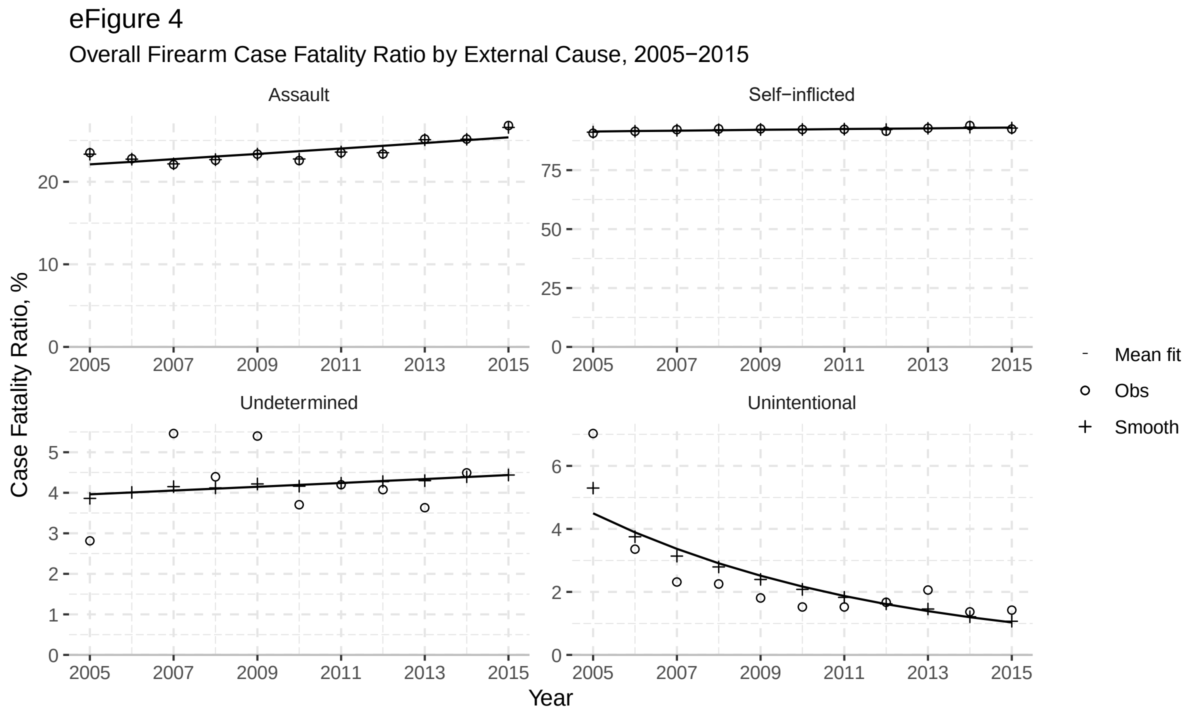

The annual overall firearm case fatality ratio between 2005 and 2015 by external cause code of injury.

© 2020 Spitzer SA et al. *JAMA Network Open.*
